# Limit to Self-Field Critical Current Density in Thin-Film, Type-II Superconductors

**DOI:** 10.3390/ma19040745

**Published:** 2026-02-14

**Authors:** Amit Goyal, Rohit Kumar, Armando Galluzzi, Massimiliano Polichetti

**Affiliations:** 1 Laboratory for Heteroepitaxial Growth of Functional Materials & Devices, Department of Chemical & Biological Engineering, State University of New York (SUNY) at Buffalo, Buffalo, NY 14260, USA; rkumar34@buffalo.edu; 2 Laboratory “LAMBDA” for Analysis of Materials Behaviour in DC and AC Fields, Department of Physics “E.R. Caianiello”, University of Salerno and CNR-SPIN Salerno, Via Giovanni Paolo II, 132, 84084 Fisciano, SA, Italy; agalluzzi@unisa.it

**Keywords:** Type-II superconductors, self-field critical current density, magnetic penetration depth, artificial pinning centers, coated conductors, critical current enhancement, thermodynamic limits in superconductors, current-carrying limits, applied superconductivity

## Abstract

In the last decade, the self-field critical current density *J*_c_(s.f.) in Type-II superconductors has been considered fundamentally limited by a Silsbee-like criterion of *J*_c_(s.f.) = *H*_c1_/λ. We show that this universal limit to self-field critical current density *J_c_*(s.f.) is not universally valid. We present several examples for this in YBa_2_Cu_3_O_7−δ_-type and REBa_2_Cu_3_O_7−δ_ thin films and one for Nb thin films and show that calculated *J_c_*(s.f.) using the Silsbee-like criterion using thermodynamic parameters has been substantially exceeded experimentally. We also show that *J_c_*(s.f.) can be significantly improved by incorporation of artificial pinning centers (APCs), further implying that no such universal limit to *J_c_*(s.f.) can exist because such an upper bound, *J_c_*(s.f.) would have to be independent of APCs. These findings call for a revision of the accepted understanding of current-carrying limits in Type-II superconductors and reveal substantial potential for improving *J*_c_ in REBCO-based coated conductors through optimization of APCs for large-scale applications, including commercial nuclear fusion.

## 1. Introduction

Kilometer-long HTS wires based on heteroepitaxial deposition of high-temperature superconductors (HTS) on single-crystal-like substrates have generated tremendous excitement for many envisioned large-scale applications [1,2,3,4,5,6,7,8,9,10]. In particular, energy generation via commercial nuclear fusion is presently of great interest and has created a flurry of interest world-wide [1,2,3,4,5,6,7,8,9,10]. In addition, there are numerous large-scale applications of HTS materials for energy transmission, energy storage and use in electric power devices in the grid. All these large-scale HTS applications, the cost, or the price/performance metric of HTS wires dictates if an application will become commercially viable. A key route to achieve this goal is by significantly increasing the performance of the HTS wires without increasing the process cost. Hence, the question of the maximum performance or the critical current density, *J_c_*, that can be achieved in a HTS wire is of paramount importance and of significant consequence to the field towards achieving conductor price/performance goals for large-scale HTS applications [11,12,13]. A well-known upper bound for the critical current density in any superconductor is the depairing current density *J_d_,* the point at which Cooper pairs are destroyed and the material transitions to a resistive, non-superconducting state. *J_d_* depends on key fundamental thermodynamic parameters such as the penetration depth, the coherence length, and on the measurement temperature. The depairing current density for Type-I superconductors is well-described by the Silsbee rule (proposed in 1917) which states that the depairing current density is the critical current density that, when flowing through the superconductor, induces a magnetic field equal to the critical field (*H_c_*) of the Type-I superconductor causing a *sharp* breakdown of the superconducting state at the critical field [14], and so *J_d_* = *H_c_*/λ, where λ is the penetration depth. Unlike Type-I superconductors, in Type-II superconductors, the transition to the normal state is gradual, starting at the lower critical field, *H_c_*_1_, where a mixed, superconducting and non-superconducting vortex state develops, and the fully resistive, non-superconducting state develops at the upper critical field, *H_c_*_2_. For most technologically relevant Type-II superconductors, *H_c_*_2_ is extremely large, whereas *H_c_*_1_ is comparatively small. Silsbee’s criterion is therefore not applicable for determining the depairing current density in Type-II superconductors.

It has been argued that while the Silsbee criterion when considering the lower critical field, *H_c_*_1_, does not result in the depairing current density (i.e., *J_d_* is not equal to *H_c_*_1_/λ), it may predict a limit to attainable *J_c_*(self-field), thereby providing an “upper limit” to possible *J_c_*(self-field) in a Type-II superconductor. Substituting the standard textbook equation for *H_c_*_1_ [15] in this formula, Talantsev and Tallon [16] obtain an expression for critical current density in thin-film, Type-II superconductors (as shown in Equation (4) of their paper):

(1) $$J_{c}=\frac{\phi_{0}}{4\pi \mu_{0}}\times \frac{\ln\left(\frac{\lambda (T)}{\xi (T)}\right)+0.5}{\lambda^{3}(T)}\;$$

where $\phi_{0}=2.06\times 10^{-15}\;Wb$, is the flux quantum, $\mu_{0}=4\pi \times 10^{-7}\frac{Wb}{Am}$, is the permeability of free space, λ= London penetration depth (nm) and ξ= coherence length (nm). The authors then examined published data in the literature on self-field *J_c_* for various thin-film superconductors, as well as the reported thermodynamic parameters (penetration depth, λ, and coherence length, ξ), and then calculated the predicted *J_c_*(s.f) obtained using Equation (1). As noted in their paper, they found no experimental case in which the measured self-field *J_c_* exceeded the value predicted by Equation (1). They therefore proposed that the *J_c_* obtained from Equation (1) represents an “upper limit” to the attainable self-field *J_c_* in thin-film superconductors whose thickness is smaller than the penetration depth λ. As stated in their paper, the authors were surprised that even for Type-II superconductors, a similar criterion, *J_c_* = *H_c_*_1_/λ, provided the “upper limit” to attainable self-field critical current density, *J_c_*(s.f.), and stated that this upper limit is valid for all Type-II superconductors and hence claimed that it was a “*universal or fundamental limit*” to attainable *J_c_*(s.f.). In their paper, it is emphasized that this fundamental limit also applies to *J_c_* for the second-generation high-temperature superconducting wires (also known as HTS 2G-wires or REBa_2_Cu_3_O_7−δ_ tapes or coated conductors) [17], which are the preferred option for high-field applications such as magnets for fusion projects [10]. Moreover, it is stated that the highest *J*_c_(s.f., *T*) will be achieved with the lowest possible value of λ, and in REBa_2_Cu_3_O_7−δ_ this can only be achieved by full oxygenation (δ→0) and removing all impurity substitution.

## 2. Methods

In this paper we report the calculation of the predicted universal or fundamental limit to J_c_(s.f.), referred to hereon as J_c_(max, s.f.)_calc._ by means of Talantsev and Tallon’s primary Equation (1) listed above [16], using reported experimental values of the fundamental thermodynamic material parameters (the London penetration depth and the coherence length) for cases approaching full oxygenation or for $\left(\delta \to 0\right)$ in REBa_2_Cu_3_O_7−δ_ and compare the obtained J_c_(s.f.) values with experimentally reported J_c_(s.f.) values. The J_c_(s.f.) is also a reference value for the J_c_ at high magnetic fields [18]. Moreover, we address the fundamental question of whether the J_c_(s.f.) can be affected by extrinsic artificial pinning centers.

First case: Using experimental values of thermodynamic parameters reported by Kiefl, R. F. et al. [19] for YBa_2_Cu_3_O_6.92_, with the effective in-plane penetration depth λ_ab_(T→0 K) ≈ 115.3 nm, and coherence length, ξ = 1.5 nm, this fundamental J_c_(s.f.) limit calculated using Equation (1) is J_c_(max, s.f., T →0 K)_calc._ ≤ 42 MA/cm^2^.

Second case: Using data from Sonier et al. [20], for an optimally doped sample of YBa_2_Cu_3_O_6.95_ with *T_c_* = 93 K and having λ*_ab_* = 112 nm, we obtain the same value for this case as for the third case highlighted below.

Third case: Using measured thermodynamic parameters λ = 112 nm and ξ = 1.6 nm reported by Stangl et al. [21], for a nearly fully oxygenated thin-film of composition YBa_2_Cu_3_O_7−δ_, the calculation for the fundamental limit or *J_c_*(max, s.f.)*_calc._*, is shown below:

$$J_{c}{\left(max,s.f.\right)}_{calc}=1.309\cdot 10^{-10}Am\times \frac{\ln\left(\frac{112\cdot 10^{-9}\;m}{1.6\cdot 10^{-9}\;m}\right)+0.5}{{\left(112\cdot 10^{-9}\right)}^{3}\;m^{3}}=1.309\cdot 10^{-10}\;Am\times \frac{4.25+0.5}{1.405\cdot 10^{-21}\;m^{3}}\;\;\;\;=1.309\cdot 10^{-10}\times 3.38\cdot 10^{21}\;\left(\frac{Am}{m^{3}}\right)\approx 4.4\cdot 10^{11}\;\frac{A}{m^{2}}=4.4\cdot 10^{7}\;\frac{A}{{cm}^{2}}=44\;\frac{MA}{{cm}^{2}}$$

## 3. Results and Discussion

**Figure 1:**
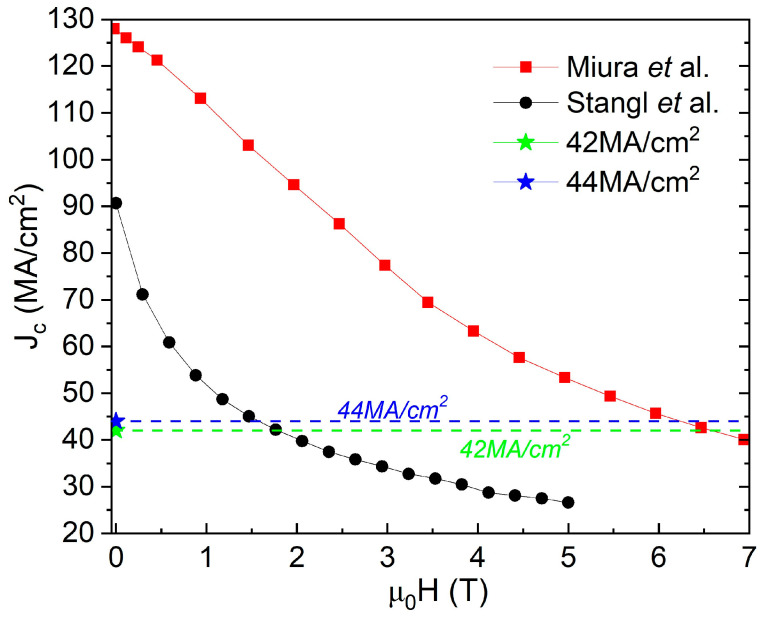
*J_c_* (H) for two films: (1) YBa_2_Cu_3_O_7−δ_ (YBCO) thin-film approaching full oxygenation from Stangl et al. [21], and (2) (Y,Gd)Ba_2_Cu_3_O_7−δ_ (YBCO) thin-film with BaHfO_3_ additions from Miura et al. [24]. Shown in the figure in blue and green is the *J_c_*(self-field) calculated using the “*Universal or fundamental limit*” per Equation (1) of 42 MA/cm^2^ and 44 MA/cm^2^ and corresponding to the first and third case in the article.

Despite the values obtained starting from the reported Equation (1), Stangl et al. [21], report an experimentally measured *J_c_*(s.f., 5 K) of ≈90 MA cm^−2^ for this undoped YBa_2_Cu_3_O_7−δ_ (YBCO) thin-film approaching full oxygenation. The plot of *J_c_* (H) at 5 K in field-range from self-field to 5 T, by Stangl [21] shown in Figure 1 clearly shows significantly higher experimentally obtained *J_c_* than that predicted by Equation (1) using values for thermodynamic parameters λ and ξ measured and reported by them for this film [16]. This *J_c_*(s.f., 5 K) is more than 100% higher than the value of 42 MA/cm^2^ (which is 44 MA/cm^2^ if λ and ξ for Stangl’s film shown above are considered) calculated using Talantsev and Tallon’s primary Equation (1). If Stangl et al.’s *J_c_*(s.f.) measurements were made at lower temperatures, approaching 0 K, the *J_c_* would have further increased manifold, even further exceeding *J_c_*(max, s.f.)*_calc._* predicted by Equation (1).

**Figure 2:**
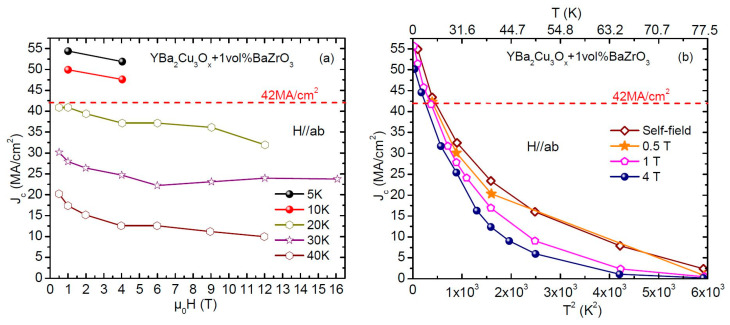
*J_c_* (H) for a PLD YBa_2_Cu_3_O_7−δ_ + 1vol%BaZrO_3_ film measured by Xu [22]: (**a**) Selected data from Figure 6.4 of Xu showing *J_c_* (H) at different temperatures, and (**b**) Selected data from Figure 6.5 from Xu showing *J_c_* (T) at different applied fields. The dashed line in both figures corresponds to the *J_c_*(max, s.f.)*_calc._* = *H_c_*_1_/λ for YBa_2_Cu_3_O_7−δ_ films, calculated by Equation (1).

The field and temperature dependences *J_c_* (H, T) of a YBCO—1 vol%BaZrO_3_ film with self-assembled columnar BZO nanorods fabricated using pulsed laser deposition (PLD), measured in transport by Xu [22,23] are shown in Figure 2. This Figure contains exactly the data presented in Figure 6.4 on page 104 and Figure 6.5 on page 105 of Xu’s thesis [22] and show that J_c_ for this film in external field at both 10 K and 5 K exceeds the theoretical limit in self-field (42 MA/cm^2^). The values of *J_c_*(s.f.) are not reported because they exceed the current limit of their transport system, which for the specific bridge geometry and film thickness was 54.6 MA/cm^2^. Even at this measurement limiting value of 54.6 MA/cm^2^, the transport *J_c_*(s.f.) is 30% higher than *J_c_*(max, s.f.)*_calc._* = 44 MA/cm^2^ for YBCO films at full oxygenation, predicted by Equation (1). In addition, if Xu et al.’s measurements were made at lower temperatures, approaching 0 K, the *J_c_* would have increased manifold, even further exceeding this “*limit*” to self-field *J_c_*. Lastly, since these YBCO films were not fully oxygenated (unlike Stangl’s films), the *J_c_*(s.f.) is expected to further rise significantly upon oxygenation to produce an overdoped state as shown by Stangl.

**Figure 3:**
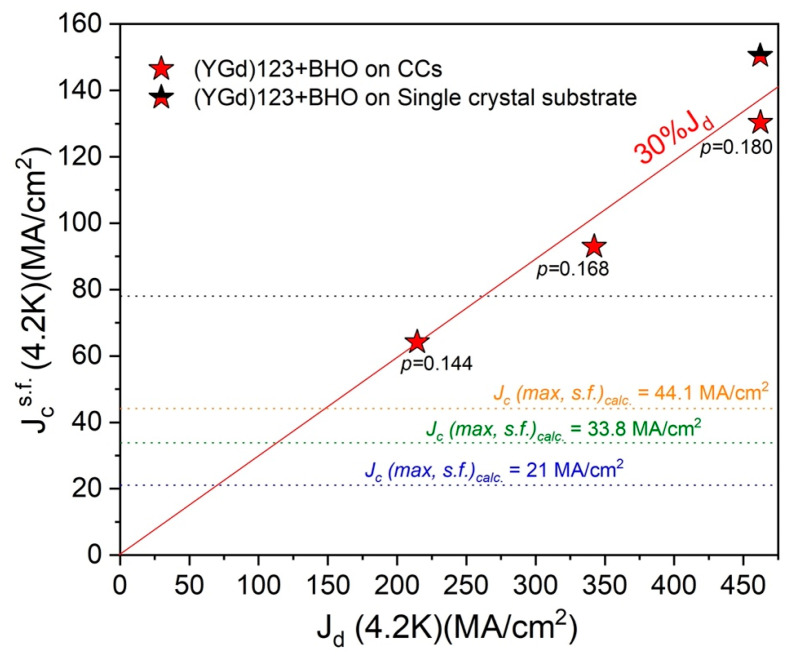
*J_c_*(s.f.) on the *y*-axis and *J_d_*(s.f) on the *x*-axis at 4.2 K for (Y,Gd)Ba_2_Cu_3_O_7−δ_ (YBCO) thin-film with BaHfO_3_ additions from Miura et al. as a function of carrier concentration, *p* [24], for *p* = 0.18, 0.168 and 0.144. The solid red line corresponds to *J_c_* equal to 30% of estimated *J_d_*. Also, shown in the plot is a dotted blue line (*p* = 0.144), a dotted green line (*p* = 0.168) and a dotted orange line (*p* = 0.18) corresponding to *J_c_(max*, *s.f.*)*_calc._* = *H_c_*_1_/λ. The highest *J_c_* at *p* = 0.18 on a CC substrate was 130 MA/cm^2^ and 150 MA/cm^2^ for a film on SrTiO_3_.

Xu et al. [22,23], also reported an experimentally measured transport *J_c_*(s.f., 4.2 K) ~55 MA/cm^2^ at 4.2 K for H//c for a thin-film superconductor of composition 15 mol.% Zr-added (Gd, Y)-Ba-Cu-O. This *J_c_*(s.f., 4.2 K) was ~30% higher than that predicted by Equation (1) of 44 MA/cm^2^. Again, if Xu et al.’s measurements were made at lower temperatures, approaching 0 K, a further pronounced increase in the value of the *J_c_* would have been obtained. Miura et al. [24] reported both a transport and magnetization *J_c_*(s.f., 4.2 K) as well as calculated *J_d_*(4.2 K) for (Y,Gd)Ba_2_Cu_3_O_7−δ_ (YBCO) thin films with BaHfO_3_ additions as a function of carrier concentration. Figure 1 shows experimentally measured *J_c_*(H) at 4.2 K in field-range from self-field to 7 T, by Miura et al. [24] showing significantly higher experimentally obtained *J_c_* than that predicted by Equation (1) (nearly 200%) using values for thermodynamic parameters λ and ξ and as discussed above. Figure 3 shows *J_c_*(s.f., 4.2 K) for three films with carrier concentration, *p*, equal to 0.18, 0.168 and 0.144. Only the film with *p* = 0.18 is overdoped and fully oxygenated. Miura et al. also provide experimentally measured values of the thermodynamic parameters, of λ and ξ, for the films as a function of carrier concentration. Table 1 provides a summary of data from Miura along with the predicted *J_c_*(max, s.f.)*_calc._* calculated using Equation (1).

As shown in Table 1, the *J_c_*(s.f., 4.2 K) is 194% higher than the “*limit*” of 44.1 MA/cm^2^ for the film on a coated conductor substrate. For the same film deposited on single-crystal, SrTiO_3_ substrate, the *J_c_*(s.f., 4.2 K) is 240% higher than this “*limit*”. For the incompletely oxygenated film corresponding to carrier concentration, *p* = 0.168, the *J_c_*(s.f., 4.2 K) is 166% higher than the predicted limit of 33.8 MA/cm^2^ for the film on a coated conductor substrate, calculated using the measured λ and ξ for this film. Lastly, for the incompletely oxygenated film corresponding to carrier concentration, *p* = 0.144, the *J_c_*(s.f., 4.2 K) is 195% higher than the “*limit*” of 21.0 MA/cm^2^ for the film on a coated conductor substrate, calculated using the measured thermodynamic parameters, λ and ξ, for this film. Again, if Miura et al.’s measurements were made at lower temperatures, approaching 0 K, the *J_c_* would have further increased by a significant margin, substantially exceeding the predicted limit to attainable self-field *J_c_*. Even for films that were not fully oxygenated with *p* = 0.168 and 0.144, the *J_c_* exceeded the predicted limit of 44.1 MA/cm^2^ for a fully oxygenated film. In Figure 3, the solid red line corresponds to *J_c_* equal to 30% of estimated *J_d_*, the dotted blue line (*p* = 0.144), the dotted green line (*p* = 0.168) and the dotted orange line (*p* = 0.18) corresponds to the *J_c_* calculated using the equation *J_c_(max*, *s.f.*)*_calc._* = *H_c_*_1_/λ. It can be seen that in all cases the experimentally obtained *J_c_* significantly exceeds the *J_c_*(*max*, *s.f.*)*_calc._* limit predicted by primary Equation (1). In fact, for all three values of the carrier concentration, the measured *J_c_* exceeds the highest *J_c_* calculated for the fully oxygenated, overdoped film shown by the orange dotted line. Figure 1 also shows *J_c_* (H, 4.2 K) for the film on a coated conductor substrate which was overdoped in comparison to the Stangl’s films and the *J_c_* (*max*, *s.f.*)*_calc._* It can be seen that the *J_c_*(s.f.) limit predicted by Equation (1) has been greatly exceeded.

If one can find a way to lower the value of the penetration depth, λ, then the “*fundamental limit*” to *J_c_*(s.f.) increases. Talantsev and Tallon [17] proposed doing so by using the lowest reported values for Gd*_x_*Y_1−*x*_Ba_2_Cu_3_O_6+*y*_ single-crystals by Perag-Barnea et al. [25] of *λ*_a_ = 103 ± 8 nm and *λ*_b_ = 80 ± 5 nm, giving an effective in-plane penetration depth of *λ*_eff_ = *λ*_ab_ = √*λ*_a_*λ*_b_ = 91 nm. Perag-Barnea et al. obtained the magnetic penetration depth by relating the number of Gd ions exposed to the microwave magnetic field to the frequency-integrated intensity of the observed ESR transitions. Combined with a value ξ = 1.5 nm, Talantsev and Tallon calculated *J*_c_(s.f., *T* →0 K) ≤ 78 MA/cm^2^. However, this value of λ_eff_ has never been experimentally confirmed in another study on Gd*_x_*Y_1−*x*_Ba_2_Cu_3_O_6+*y*_ single-crystals or reported for any Gd*_x_*Y_1−*x*_Ba_2_Cu_3_O_6+*y*_ film on a coated conductor substrate or on a single-crystal substrate such as SrTiO_3_. Regardless, even using this value of 78 MA/cm^2^ as the fundamental limit to *J_c_*(s.f.) for Gd*_x_*Y_1−*x*_Ba_2_Cu_3_O_6+*y*_ films, the *J*_c_ is exceeded by 67% for a film on a coated conductor substrate and as shown in Figure 3 and Table 1. Figure 3 shows a dotted black line at 78 MA/cm^2^. The experimentally measured *J*_c_ exceeds this value by ~15% for *p* = 0.168, by ~67% for *p* = 0.18 for films on a coated conductor substrate and by ~92% for a film on a single-crystal substrate with *p* = 0.18.

**Figure 4:**
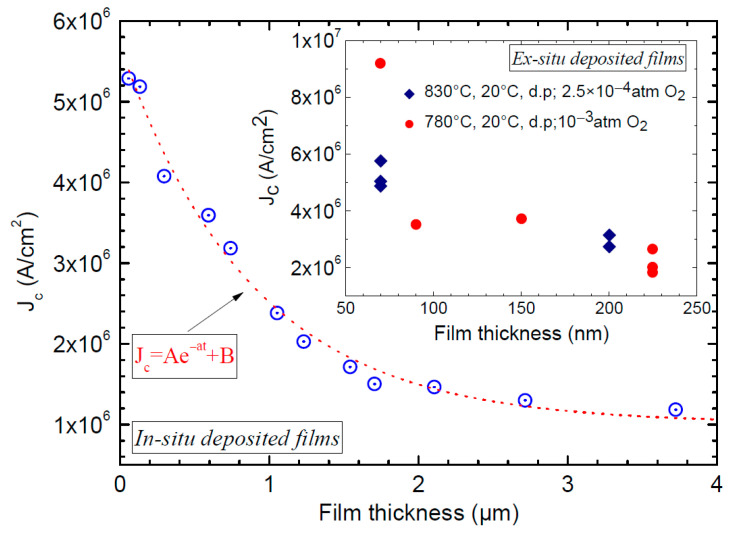
J_c_(s.f.) of thin films increases exponentially with decrease in thickness, for in situ PLD films [28] and also for ex situ films as shown in the inset [27]. In both cases, the J_c_(s.f.) increases exponentially when film thickness is much smaller than the typical REBCO penetration depth.

In their paper [16], the authors argued that their universal self-field *J_c_* limit applies only to films with thicknesses smaller than λ, since films thicker than λ allow flux entry from the edges, implying that the proposed universal limit would no longer hold in such cases. However, this consideration becomes relevant only if the thickness dependence of *J_c_* in REBCO films were such that *J_c_* continued to increase for thicknesses exceeding λ. In contrast, experimental evidence shows that the self-field *J_c_* increases rapidly at small film thicknesses [26,27,28], and in most cases grows exponentially for thicknesses below the penetration depth. This is clearly shown in Figure 1 of Matsui et al. [26], Figure 9 of McIntyre et al. [27] and Figure 3 in Foltyn et al. [28]. McIntyre et al. [27] reported on films made by chemical solution deposition with thicknesses ranging from 70 nm to 300 nm and Foltyn et al. [28], reported on films made using pulsed laser ablation with thicknesses ranging from 65 nm to 6.4 μm, and in both cases, the *J_c_*(s.f.), increased exponentially at lower film thicknesses below 150 nm as shown in Figure 4 for in situ PLD films upto 4 μm in thickness. In fact, it is well appreciated that the high self-field *J_c_* of very thin films is due to a dense array of misfit dislocations at the film/substrate interface, raising the self-field *J_c_* of such very thin films [29,30,31], with the implication that these defects are causing the *J_c_*(s.f.) to be increased. Given this trend of the thickness dependence of *J_c_*(s.f.), it can be reasonably expected that *J_c_*(s.f., 4.2 K) of films reported in Xu et al., Stangl et al. and Miura et al. will further increase quite substantially at lower film thicknesses, even more significantly surpassing the presumed universal or fundamental limit of self-field *J_c_*.

**Figure 5:**
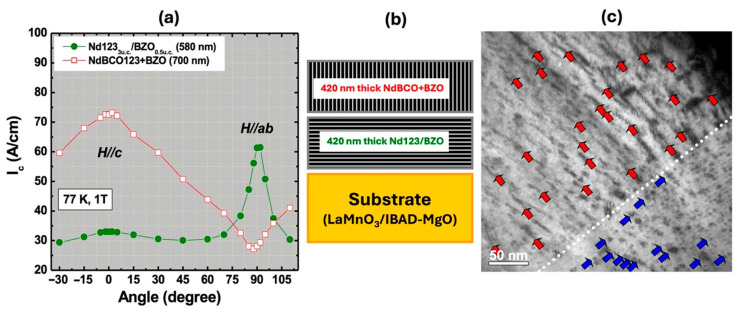
Hybrid NdBa_2_Cu_3_O_x_ + BaZrO_3_ (BZO) films, with a total thickness of 0.86 μm, deposited using PLD on a coated conductor substrate [39]. The bottom half of the film has BZO nanorods aligned parallel to the substrate surface or parallel to the ab-plane of NdBa_2_Cu_3_O_x_, and the top half of the film with BZO nanorods aligned perpendicular to the substrate surface or parallel to the c-axis of NdBa_2_Cu_3_O_x_. (**a**) Angular dependence of *I_c_* at 77 K, 1 T, showing that pinning corresponds to the orientation of BZO. (**b**) Schematic of film cross-section showing alignment of BZO in film and (**c**) high-resolution transmission electron microscopy image showing alignment of BZO nanostructures. The dashed white line separates the two halves of the film with the blue and red arrows pointing to BZO nanostructures in bottom and top halves of the film.

For extreme Type-II superconductors such as REBa_2_Cu_3_O_7−δ_ (REBCO), where *H_c_*_2_ is very high (150 T for optimally doped YBCO [32]) and *H_c_*_1_ is very low (~34–35 mT for optimally doped YBCO [33]), the self-field generated by the current exceeds *H_c_*_1_ even at low currents and the sample enters the mixed state where the *J_c_* is then determined by vortex-pinning. Hence, it is not surprising that a self-field *J_c_* calculated using the formula, *J_c_* = *H_c_*_1_/λ, was experimentally exceeded. In reference [16], the authors claim that self-field *J_c_* is fully independent of pinning. They noted a single exception reported by Schindler et al. [34], where neutron irradiation increased the self-field *J_c_* from 19.4 to 32 MA/cm^2^. However, this result was ruled out on the grounds that Schindler et al. employed a high electric-field criterion. It is correct that artificial pinning centers (APCs) generated via irradiation after film growth can in most cases lower *J_c_* (s.f.) due to a loss of effective cross-section area and/or damage to the two-dimensional CuO_2_ planes. However, there are many additional studies in the literature that show the *J_c_* (s.f.) to increase very substantially with introduction of APCs. In contrast to post-growth irradiation, where APC formation is decoupled from the film-growth process, non-irradiative methods for introducing APCs directly influence film growth, morphology, microstructure, crystallographic texture, and epitaxy, and in most cases lead to a significant enhancement of the *J_c_* (s.f.). Here, we cite only a few studies showing clear increase in *J_c_* (s.f.) via introduction of APCs—see Figure 4 of Miura et al. [24], Figure 4 of Diez-Sierra et al. [35], Figure 3 of Vaimala et al. [36], Figure 2 of Jha et al. [37] and Figure 4 of Jha et al. [38]. As mentioned previously, this can also potentially be a secondary effect due to improved film microstructure, morphology, texture, etc., with the introduction of APCs. In Wee et al. [39], defect engineering resulted in a massive increase in transport *J_c_*(s.f., 77 K), from 1.4 MA/cm^2^ to 2.5 MA/cm^2^ (~78% increase in *J_c_*(s.f.)) for a film of NdBa_2_Cu_3_O_7−δ_ with the incorporation of engineered, non-superconducting, nanoscale APCs compared to a NdBa_2_Cu_3_O_7−δ_ with no engineered APCs incorporated. Figure 5 shows that for this film, 50% of the film was nanostructured such that the BaZrO_3_ (BZO) nanorods were aligned perpendicular to the film and for the other 50% of the film, the BZO nanorods were aligned parallel to the c-axis of YBCO [39]. This film had a combined thickness of 0.86 μm.

**Figure 6:**
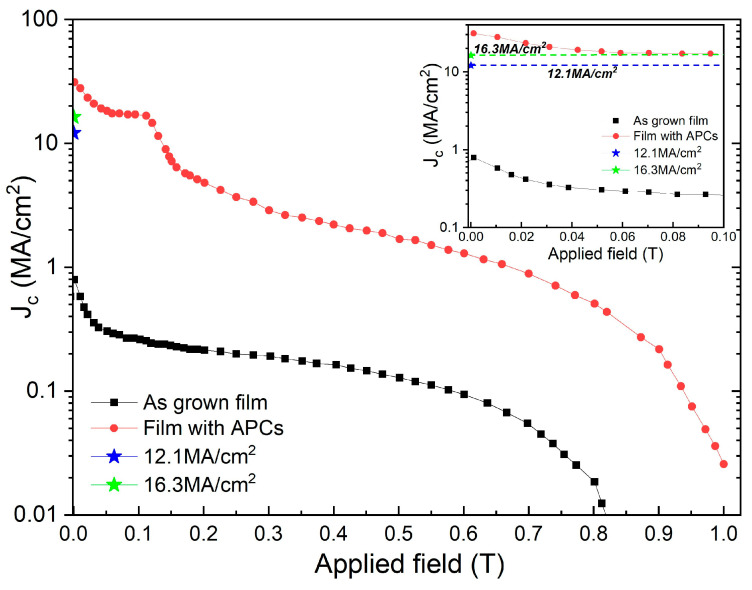
*J_c_* (H) at 5K for a Nb thin-film with and without APCs from Dinner et al. [40]. The black points and line correspond to *J_c_* for the film with no APCs. The red points and line correspond to *J_c_* for the film with nanoscale APCs. Also shown in the figure with a blue and green asterisk are *J_c_* values calculated using Equation (1) for coherence length, ξ, of 23.1 nm and 40 nm, respectively. Inset: a magnification in the range 0 T and 0.1 T is shown to better visualize the higher values of the experimentally obtained self-field *J_c_* for the film with APCs (red points) than the ones calculated using the so-called predicted maximum attainable self-field *J_c_* using expression, *J_c_(max*, *s.f.*)*_calc._* = *H_c_*_1_/λ.

While the available data for HTS materials other than REBCO is limited, we further show that this universal or fundamental limit to *J_c_*(s.f.) also does not apply to other Type-II superconductors. We consider Nb, a classical Type-II superconductor. Dinner et al. reported achievement of extremely high critical current density in Nb thin films via through-thickness arrays of APCs via ion-milling through anodized aluminum oxide thin- film templates [40]. They reported experimentally measured transport *J_c_* (H) in Nb thin films with optimized APCs (in terms of size and spacing) to be 50 times greater than Nb film with no APCs at self-field [40]. In fact, Figure 6 shows *J_c_* (H) at 5.0 K for a Nb thin-film with and without APCs from Dinner et al. [40] with the field applied perpendicular to the film surface. The black points and line correspond to *J_c_* for the film with no APCs. The red points and line correspond to *J_c_* for the film with nanoscale APCs. The transport *J_c_*(s.f., 5 K) of the Nb film with no APCs or pinning centers was ~0.6 MA/cm^2^ and the transport *J_c_* (s.f., 5 K) of the Nb film with optimized, through thickness APCs was 30 MA/cm^2^. This further clearly demonstrates that in Type-II superconductors, *J_c_*(s.f.) is also dependent on pinning. Therefore, our analysis shows that (Y,Gd)Ba_2_Cu_3_O_7−δ_ (YBCO) thin films with BaHfO_3_ and through-thickness arrays of APCs via ion-milling through anodized aluminium oxide thin-film templates in Nb thin films were both extremely effective in increasing self-field *J_c_*.

The penetration depth, λ, for these films was 120 nm [40,41] and the coherence length, ξ(5 K) = 40 nm [41]. However, since, from Ginzburg-Landau theory, ${\xi \left(T\right)=\xi }_{0}{\left(1-\frac{T}{T_{c}}\right)}^{-1/2}$, with T_c_ = 7.5 K (from Dinner et al., page 4), it results ξ_0_ = 23.1 nm at T = 0 K. Based on these thermodynamic parameters, Table 2 shows the calculated values of *J_c_*(max, s.f.)_calc._ using the primary equation mentioned above corresponding to *J_c_(max*, *s.f.*)*_calc._* = *H_c_*_1_/λ.

In Figure 6, the blue and green asterisk correspond to these *J_c_* values calculated using the equation reported in reference [16], for coherence length, ξ, of 23.1 nm (@ 0 K) and 40 nm (@ 5 K), respectively. As shown in Table 2 above, the experimentally obtained transport *J_c_*(s.f.) of 30 MA/cm^2^ is ~100% higher than that predicted by [16]. The inset of Figure 6 clearly shows the experimentally obtained self-field *J_c_* compared to that calculated using the so-called predicted maximum attainable self-field *J_c_* using expression, *J_c_* (*max*, *s.f.*)*_calc._* = *H_c_*_1_/λ. The transport measurements reported in Dinner et al. [40] were made on 12 μm wide bridges, wide enough for the geometrical issues to be not relevant. A validation for this stems from the fact that for same bridge width, the Nb film with no APCs only had a transport *J_c_*(s.f.) of ~0.6 MA/cm^2^.

Although Xu [22], published in 2012, reported transport *J_c_* of YBCO thin films on CC substrates, Xu et al. [23], published in 2014, reported transport *J_c_* of (Y,Gd)BCO thin films on CC substrates, and Dinner et al. [40], published in 2011, reported transport *J_c_* of Nb thin films, these references were not considered in reference [16]. In addition, significant enhancements to transport *J_c_*(s.f.) of YBCO thin films on CC substrates via incorporation of APCs have been shown recently in many reports [21,22,23,24,35,36,37,38,39,40]. These references now cast strong doubt on the premise of a universal or fundamental limit to *J_c_*(s.f.) for Type-II superconductors given by the expression, *J_c_(max*, *s.f.*)*_calc_* = *H_c_*_1_/λ, as their primary equation.

In short, we have shown the following:

(1) Experimentally obtained, *J_c_*(s.f.), significantly higher than that calculated using Talantsev and Tallon’s primary Equation (1) [16] have been reported (by Xu et al. [22,23], Stangl et al. [21] and Miura et al. [24]) for YBa_2_Cu_3_O_7−δ_-type and REBa_2_Cu_3_O_7−δ_ thin films and by Dinner et al. [40] for Nb films, rendering predictions of *J*_c_(s.f.) by a Silsbee-like criterion to be incorrect. A summary of the samples, materials, parameters, and self-field critical current densities considered in this work is reported in Table 3:
(2) Due to the established, rapid decrease in the critical current density with thickness, for films thinner than the films reported in point (1) above, we expect an even higher *J_c_*(s.f.) further exceeding the *J_c_* (*max*, *s.f.*)*_calc._* limit predictions, and
(3) *J_c_*(s.f.) can be significantly increased with introduction of extrinsic artificial pinning centers [35,36,37,38,39,40], further rendering the *J_c_(max*, *s.f.*)*_calc._* limit using a Silsbee-like criterion invalid.

In an alternative analysis, Mastushita and Kiuchi estimated the upper limit of achievable critical current density via optimization of APCs under the influence of kinetic energy using Ginzburg-Landau theory [42]. They estimated the upper limit of the achievable *J_c_* to be 67% of the depairing current density in the London limit [42]. They argue that the strong flux pinning by self-assembled nanorods when optimized, could provide such high *J_c_* approaching this upper limit of 67% of *J_d_* [42]. Self-assembled nanorods or nanocolumns consisting of perovskite (BaMO_3_) and double perovskite (Ba_2_RETaO_6_ and Ba_2_RETaO_6_) [43,44,45,46,47,48,49,50,51,52,53,54,55,56] nanorods have been shown to form controllable self-assembled microstructures with greatly enhanced *J_c_* and flux-pinning. Very high-*J_c_* has also been achieved in microstructures comprising randomly oriented nanoparticles of Y_2_BaCuO_x_ [57], BaZrO_3_ [58,59,60,61] and RE_2_O_3_ [62,63]. A critical-current-by-design paradigm to tailor and enhance *J_c_* using time-dependent Ginzburg-Landau simulations coupled with Monte-Carlo and molecular dynamics has been proposed to further optimize *J_c_* [11]. The data collectively demonstrate that J_c_(s.f.) is a highly tunable quantity governed by microstructural design, suggesting that optimization strategies rather than fixed limits should guide practical materials development, and that further opportunities are present for theorists to develop deeper physical insights to more accurately predict a limiting *J_c_*(s.f.) in thin-film Type-II superconducting films.

## 4. Conclusions

It was shown that the “*universal or fundamental limit*” to self-field *J_c_* calculated using a Silsbee-like criterion of *J_c_(max*, *s.f.*)*_calc._* = *H_c_*_1_/λ, for Type-II superconductors is not valid. This was established via detailed calculations for several YBa_2_Cu_3_O_7−δ_-type and REBa_2_Cu_3_O_7−δ_ thin films and then comparing the obtained *J_c_(max*, *s.f.*)*_calc._* values to experimental *J_c_*(s.f.) values. This was also shown to be the case for another Type-II superconductor, Nb, where experimental *J_c_*(s.f.) was found to be significantly higher than the predicted fundamental limit calculated via the equation, *J_c_*(*max*, *s.f.*)*_calc._* = *H_c_*_1_/λ, using reported thermodynamic parameters for the film. In addition, it was established that in Type-II superconductors, the *J_c_*(s.f.) also depends on extrinsic pinning centers, a key fundamental question in the field. The fact that *J_c_*(s.f.), in superconductors with different substrates, can be significantly improved by incorporation of artificial pinning centers (APC’s), further implies that no universal limit to *J_c_*(s.f.) can exist because for such a universal and fundamental limit to exist, *J_c_(max*, *s.f.*)*_calc._* would have to be independent of extrinsic pinning centers and different pinning mechanisms.

This leads to an important conclusion that the performance of REBa_2_Cu_3_O_7−δ_ based thin-film coated conductors have not reached their limit to attainable *J_c_*(s.f.). Since the depairing current density in (Y,Gd)Ba_2_Cu_3_O_7−δ_ (YBCO) thin films with BaHfO_3_ is still a factor of three to four higher than the highest experimentally achieved *J_c_*, the potential headroom for increasing *J_c_* is still enormous. There remains substantial room for enhancement of *J_c_* of commercial coated conductors which could significantly improve the price-to-performance ratio of high-temperature superconducting wires. Such progress would directly accelerate the deployment of superconductors across large-scale technologies, including energy generation (commercial nuclear fusion), transmission (superconducting ac and dc cables), and storage (superconducting magnetic energy storage systems, SMES), as well as in energy-efficient devices such as motors, generators, and fault-current limiters. Given the recent excitement generated due to the possibility of commercial nuclear fusion enabled by coated conductor HTS wires, this is an *extremely important and key point* to be considered in scaling-up fabrication of HTS wires.

## Figures and Tables

**Table 1:** Thermodynamic parameters, λ and ξ, *J_c_*(s.f.) at 4.2 K, *J_d_* (0 K) from Miura et al. [24], and *J_c_*(*max*, *s.f.*)*_calc__._* calculated using the equation, *J_c_(max*, *s.f.*)*_calc__._* = *H_c_*_1_/λ [16] for (Y,Gd)Ba_2_Cu_3_O_7−δ_ (YBCO) thin films with BaHfO_3_ additions, at different carrier concentration, *p*.

Carrier Concentration, *p*	London Penetration Depth, λ_ab_ (nm)	Coherence Length, ξ_ab_ (nm)	Experimentally Measured Transport J_c_(s.f., 4.2 K) (MA/cm^2^)	Estimated J_d_(0) (MA/cm^2^)	$\mathrm{Calculated}\;J_{c}(\max,\;s.f.){\mathrm{calc}}_{.}\;=\;Hc_{1}/\lambda$ Using Equation (1) from Reference [16]
0.18 (CC subs)	112	1.61	130	498.02	44.1
0.18 (SrTiO_3_)	112	1.61	150	498.02	44.1
0.168	122	1.84	90	367.68	33.8
0.144	143	2.14	62	230.57	21.0

**Table 2:** Thermodynamic parameters, λ and ξ from Dinner et al. [40], and *J_c_*(max, s.f.)_calc._ calculated using the equation, *J_c_(max*, *s.f.*)*_calc._* = *H_c_*_1_/λ [16] for Nb thin films.

λ(0) (nm)	ξ (nm)	Measured *J_c_(s.f.*, 5 K) (MA/cm^2^)	*J_c_(max*, *s.f.*)*_calc._* (MA/cm^2^)
120	40 (@ 5 K)	30	12.1 (@ 5 K)
120	23.1 (@ 0 K)	30	16.3

**Table 3:** Summary of samples and self-field critical current density results.

Material/Sample	APCs Type and Morphology	λ (nm)	ξ (nm)	T (K)	Calculated J_c_(max,s.f.) (MA/cm^2^)	Experimental J_c_(s.f.) (MA/cm^2^)	Increment Relative to the Theoretical Limit
YBCO (Stangl)	Pure with no APC’s, near full oxygenation	112	1.6	5	≈44	≈90	+104%
YBCO + 1% BZO (Xu)	Columnar BaZrO_3_ nanorods	112	1.6	5, 10	≈44	≥54.6	≥+24%
15 mol.% Zr-added (Gd, Y)-Ba-Cu-O (Xu)	Columnar BaZrO_3_ nanorods	112	1.6	4.2	≈44	55	+25%
(Y,Gd)BCO + BHO (Miura, *p* = 0.18, CC)	Columnar BaHfO_3_ nanorods	112	1.61	4.2	44.1	130	+194%
(Y,Gd)BCO + BHO (Miura, *p* = 0.18, STO)	Columnar BaHfO_3_ nanorods	112	1.61	4.2	44.1	150	+240%
(Y,Gd)BCO + BHO (Miura, *p* = 0.168)	Columnar BaHfO_3_ nanorods	122	1.84	4.2	33.8	90	+166%
(Y,Gd)BCO + BHO (Miura, *p* = 0.144)	Columnar BaHfO_3_ nanorods	143	2.14	4.2	21.0	62	+195%
Nb thin-film (Dinner)	Columnar nanoholes	120 120	40 (@ 5 K) 23.1 (@ 0 K)	5 5	12.1 16.3	30 30	+148% +84%

## Data Availability

The original contributions presented in this study are included in the article. Further inquiries can be directed to the corresponding authors.
